# Differentiation of *Lupinus* and *Mimosa* by Machine Learning Employing Spectroscopic Data: A Comparative Study

**DOI:** 10.1021/acsomega.5c13597

**Published:** 2026-04-24

**Authors:** Mariana Koetz, Maria H. Vendruscolo, Andressa K. Maia, Stefanie L. K. Martinelli, Marina González, Milton K. Sasaki, Sílvia T. S. Miotto, Marina Scopel, Elen de Oliveira Alves, Rodrigo M. Viegas, Alexandre M. Fuentefria, Marco F. Ferrão, José A. S. Zuanazzi

**Affiliations:** † Faculty of Pharmacy, Center for the Study of Natural Products, Federal University of Rio Grande do Sul, Porto Alegre, RS 90610-000, Brazil; ‡ Institute of Chemistry, Federal University of Rio Grande do Sul, Porto Alegre, RS 91501-970, Brazil; § Institute of Biosciences, Department of Botany, Federal University of Rio Grande do Sul, Porto Alegre, RS 91501-970, Brazil; ∥ Faculty of Pharmacy, Federal University of Minas Gerais, Belo Horizonte, MG 31270-901, Brazil; ⊥ Faculty of Pharmacy, University of Porto, Porto 4050-313, Portugal; # Secretariat of Information and Communication Technology, Federal University of Health Sciences of Porto Alegre, Porto Alegre, RS 90050-170, Brazil

## Abstract

This study investigated the complex chemical composition of species from the genera *Lupinus* and *Mimosa* (Fabaceae) from southern Brazil using ultraviolet–visible (UV–vis) and Fourier transform infrared (FTIR) spectroscopy, in conjunction with machine learning techniques. The results revealed that, despite similarities in the presence of secondary metabolites such as flavonoids and alkaloids, the genera can be accurately distinguished. To overcome the high dimensionality and collinearity of spectral data, linear discriminant analysis (LDA) was coupled with variable selection by successive projection algorithm (SPA) and genetic algorithm (GA). These models successfully isolated specific spectral markers, achieving 100% sensitivity, specificity, and accuracy in sample classification. This study highlights the effectiveness of combining accessible spectroscopic techniques with machine learning in plant taxonomy, offering a robust preliminary alternative for species differentiation and enhancing the understanding of chemical diversity within the Fabaceae family.

## Graphical abstract

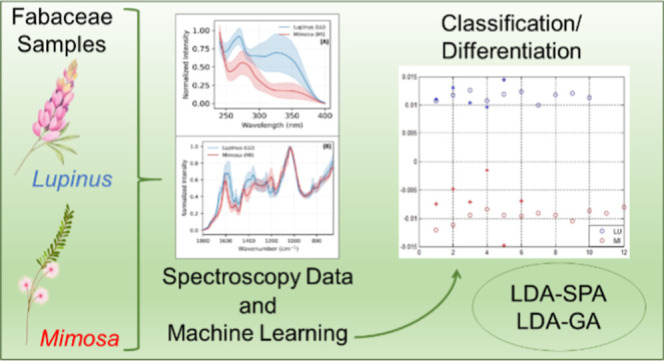

## 1 Introduction

The Fabaceae (Leguminosae) family is one of the largest among Angiosperms, comprising approximately 795 genera and almost 20,000 species, distributed throughout the world, mainly in subtropical and tropical regions.^1–3^ This group stands out for its richness in bioactive compounds and its ecological and economic importance, notably including plants that establish symbiosis with nitrogen-fixing bacteria in the soil.^4–6^

Species of the Fabaceae family possess several compounds, including alkaloids, flavonoids, terpenoids, tannins, and saponins, which have been investigated for their pharmacological properties, such as anti-inflammatory, antioxidant, and antimicrobial activities.^7–12^

The genera *Lupinus* L. and *Mimosa* L. (Fabaceae) are notable for the complexity of their chemical profiles and potential applications of their bioactive compounds. *Lupinus lanatus* Benth. and *Lupinus luteus* L. are widely studied due to the presence of quinolizidine alkaloids. These alkaloids exhibit significant pharmacological properties, including analgesic, antiarrhythmic, and antitumor effects.^13,14^ Furthermore, species of the genus *Lupinus*, owing to the ability of legumes to fix atmospheric nitrogen, are also used as sources of protein and play a fundamental role in sustainable agriculture, contributing to soil recovery.^15,16^

Similarly, species of the genus *Mimosa* present diverse bioactivities and therapeutic potential. Martínez-Higuera et al.^17^ reported that a hydrogel with silver nanoparticles synthesized with the addition of *Mimosa tenuiflora* (Willd.) Poir. demonstrated bactericidal and anti-inflammatory effects, accelerating the recovery of burns. Valencia-Gómez et al.^18^ also observed that O-carboxymethyl chitosan films enriched with *M. tenuiflora* extract maintain their physicochemical properties and promote rapid healing by stimulating inflammation and fibroblast proliferation. Furthermore, studies with *Mimosa pigra* L. extracts have demonstrated the ability to inhibit the proliferation of WEHI-3 cells, contributing to the treatment of acute myeloid leukemia,^19^ while Nguyen et al.^20^ highlighted the pulmonary antifibrotic potential of this genus, protecting alveolar epithelial cells and fibroblasts against inflammatory injuries during lung fibrogenesis.

The immense chemical complexity and taxonomic diversity within the Fabaceae family demand rigorous analytical strategies to fully characterize their metabolite profiles, ensure quality control of their bioactive extracts, and resolve taxonomic ambiguities. To achieve these scientific objectives, researchers have increasingly relied on comprehensive chemical fingerprinting and metabolomics. Consequently, several species of this family have been extensively studied using a variety of advanced analytical techniques—such as NMR, flame atomic absorption spectrometry (F-AAS), GC–MS, LC–MS, HPLC–MS/MS, UPLC-QTOF-MS, and capillary electrophoresis—for the classification of chemical profiles and data processing using chemometric methods. For instance, high-performance thin-layer chromatography was used to compare the fingerprint of propolis samples with those of plant bud extracts and, through palynological analysis, identified that the pollen grains of the propolis samples belonged to the families Asteraceae, Fabaceae, Rosaceae, among others.^21^ HPLC was used for chemometric studies and phenolic metabolomics of *Cassia absus* seeds L.^22^ F-AAS was used to classify the elemental profiles of legumes and oilseeds.^23^ The contents of oligosaccharides of the raffinose and sucrose families in *Brassica* L., *Lupinus*, *Pisum* L., and *Hordeum* L. species were investigated by chemometric principal component analysis (PCA) using capillary electrophoresis.^24^ Furthermore, GC–MS, LC–MS, and 1D NMR techniques were used to compare the metabolite profile and fingerprint of 25 compounds from four species (*Glycyrrhiza echinata* L., *Glycyrrhiza glabra* L., *Glycyrrhiza inflata* Batalin, and *Glycyrrhiza uralensis* Fisch ex DC.).^25^ The species *Sutherlandia frutescens* (L.) R. Br. and *Sutherlandia microphylla* Burch ex DC. were compared to establish phytochemical variations and chemometric analysis using UPLC-TQ-MS/MS.^26^

Although these advanced analytical methods are capable of providing detailed chemical profiles and accurate structural information, which enable the identification and quantification of a wide variety of bioactive compounds, they have the disadvantages of requiring expensive equipment, specialized maintenance, and trained operators, thereby limiting the accessibility of these techniques and rendering them unfeasible for laboratories with more restricted resources.^27–29^

To overcome the limitations of high-cost equipment and provide a scalable alternative for species differentiation, this study proposes the use of Fourier transform infrared (FTIR) and ultraviolet–visible (UV–vis) spectroscopy. The fundamental hypothesis motivating the choice of these complementary techniques is that the distinct secondary metabolite profiles of *Lupinus* (e.g., quinolizidine alkaloids) and *Mimosa* (e.g., diverse flavonoids and tannins) will produce unique and discriminative spectral signatures. Specifically, UV–vis spectroscopy is highly sensitive to conjugated π-systems, making it an excellent tool for detecting variations in phenolic compounds and flavonoids. Concurrently, FTIR spectroscopy provides a comprehensive vibrational fingerprint of molecular functional groups, such as carbonyls and aromatic rings, which are essential for identifying alkaloid and phenolic backbones. Previous studies have demonstrated that these techniques, in addition to being simpler and highly accessible, exhibit good reproducibility and provide robust chemical information.^30,31^ The possibility of associating these specific spectral signatures with machine learning algorithms allows the generated data to be used to build robust predictive and classification models,^32,33^ enabling the identification of underlying chemical patterns and facilitating the reliable separation of different genera and species.^30,34^

Chemometric methods encompass machine learning techniques that enable the analysis and interpretation of complex chemical data and are divided into supervised and unsupervised methods, each with specific applications aimed at optimizing information extraction.^35,36^

Among the unsupervised methods, we highlight PCA and hierarchical cluster analysis (HCA). These methods are particularly useful for identifying patterns in data sets. PCA reduces the dimensionality of the data, highlighting the main patterns and variables while preserving as much of the original information as possible. HCA groups samples are based on similarities and are used for classification purposes.^35^

In complex plant matrices, such as those of the Fabaceae family, spectral data often exhibit high dimensionality and severe band overlapping. While conventional exploratory chemometrics (like PCA) can reveal data structure, they are often insufficient for identifying the specific, nonobvious spectral features responsible for species differentiation. Machine learning algorithms, particularly those coupled with variable selection like successive projection algorithm (SPA) and genetic algorithm (GA), overcome this by isolating the most discriminant spectral variables, eliminating noise and redundancy, and providing deeper insights into the specific chemical markers driving the classification.^21,35,36^

Supervised methods, which require prior information about sample classes, include linear discriminant analysis (LDA), in which the SPA and GA are prominent. SPA is used to efficiently select relevant variables, minimizing redundancy and improving the accuracy of predictive models, while GA, inspired by biological evolution, randomly selects new individuals in the current population at each repetition.^36,37^ The application of these methods to natural product data facilitates the classification and discrimination of bioactive compounds, aiding in the differentiation between species and understanding their chemical and biological properties.

Given the above, this study aims to investigate the genera *Lupinus* and *Mimosa* using UV/vis and FTIR techniques in conjunction with machine learning in order to develop predictive models based on chemical data from samples collected in Rio Grande do Sul (Brazil), effectively classifying and differentiating the genera.

## 2 Materials and Methods

### 2.1 Reagents and Materials

The following chemical reagents were used to obtain the extracts: methanol and ethanol (Neon, Suzano, SP, Brazil); ethyl ether (Dinâmica, Indaiatuba, SP, Brazil); and distilled water (GFL 2008 Water Distiller).

### 2.2 Plant Material

Samples of *Lupinus* (*n* = 15) and *Mimosa* (*n* = 18) were collected in Rio Grande do Sul, Brazil (Table 1), identified, and their voucher specimens were deposited in the ICN-UFRGS Herbarium. The samples were dried and ground in a knife mill, and their particle size was standardized to 500–710 μm.

**Table 1:** Set of Plant Species Collected in Rio Grande do Sul, Brazil

*Mimosa*			*Lupinus*		
code	species	collection number	code	species	collection number
MI01	sp1	205,639	LU01	*albescens*	205,647
MI02	sp3	205,672	LU02	*bracteolaris*	205,608
MI03	sp4	205,673	LU03	*bracteolaris*	205,611
MI04	*dolens*	205,862	LU04	*bracteolaris*	205,636
MI05	*bifurca*	205,675	LU05	*bracteolaris*	205,652
MI06	*baptistae*	205,650	LU06	*bracteolaris*	205,660
MI07	*glycyrrhizoides*	205,858	LU07	*gibertianus*	205,668
MI08	*ramurosa*	205,635	LU08	*guaraniticus*	205,872
MI09	*dutrae*	205,670	LU09	*lanatus*	205,609
MI10	*cruenta*	205,642	LU10	*lanatus*	205,644
MI11	*daleoides*	205,850	LU11	*lanatus*	205,654
MI12	*myriophylla*	205,884	LU12	*multiflorus*	205,630
MI13	*pilulifera*	205,640	LU13	*paranensis*	205,631
MI14	*riverensis*	205,847	LU14	*paranensis*	205,633
MI15	*schleidenii*	205,665	LU15	*multiflorus*	205,883
MI16	*bimucronata*	210,936			
MI17	*pigra*	210,935			
MI18	*pudica*	–^a^			

a Exsiccata not yet deposited.

### 2.3 Preparation of Lyophilized Extracts

The metabolites were extracted with analytical grade methanol at a drug-to-solvent ratio of 1:20 using four 20 min cycles of ultrasonic extraction. The resulting solution was filtered, and the filtrate was collected in a round-bottom flask. The solvent was removed by evaporation using a rotary evaporator. The dried extract was resuspended in 30 mL of water, and chlorophyll was removed by four cycles of washing with 20 mL of diethyl ether. The aqueous phase was frozen and lyophilized. The lyophilized extracts were used in the experiments.

### 2.4 Ultraviolet–Visible (UV–Vis) Spectrophotometry Analysis

Lyophilized extracts were prepared as solutions of 10 μg/mL in 80% ethanol. These solutions were prepared in triplicate and analyzed using an Agilent Technologies 8453 UV–vis spectrophotometer in scanning mode (240–400 nm). The resulting visible spectral data were exported as a CSV file, and the numerical data matrix was used for multivariate analyses.

### 2.5 Fourier Transform Infrared Spectroscopy (FTIR) Analysis

Lyophilized extracts were analyzed in duplicate using an Agilent Technologies Cary 630 FTIR spectrometer with a zinc selenide (ZnSe) crystal and helium/neon laser. Spectra were generated in the region of 1800–600 cm^–1^, with 32 scans and a resolution of 4 cm^–1^. The resulting numerical data matrix was used for multivariate analyses.

### 2.6 Multivariate Analysis

#### 2.6.1 Software

ChemoStat V2 was used for the HCA and PCA. MATLAB software, version 2014a (The MathWorks Inc., MA, USA), was used for LDA classification models, employing variable selection with GA and SPA. These models were developed using VS TOOLBOX, a proprietary toolbox software developed by the Analytical Instrumentation and Chemometrics group.^38^

#### 2.6.2 Unsupervised Methods

Exploratory analyses using HCA and PCA were applied to the UV–vis and FTIR data to reveal similarities between the samples. Data preprocessing is a critical step in minimizing instrumental noise and scattering effects before multivariate analysis. In this study, preprocessing included Savitzky–Golay smoothing (second-order polynomial, window size = 13 points), which was empirically selected to adequately remove high-frequency instrumental noise without oversmoothing or distorting the relevant spectral absorption bands. Subsequently, baseline correction and normalization were performed using min–max normalization (range 0–1) applied across the full spectral region of each individual sample. Finally, the data were globally mean-centered across the entire data set to ensure that all variables were evaluated based on their variance relative to the overall mean. Regarding data integrity, a rigorous visual and exploratory inspection of the spectral data and PCA scores was established to assess the presence of potential outliers prior to model training.

#### 2.6.3 Supervised Methods

The direct application of classical LDA to full-spectrum data is generally unfeasible due to the high degree of collinearity and the fact that the number of variables greatly exceeds the number of samples, which inevitably leads to model overfitting and matrix singularity. To overcome this limitation and construct chemically interpretable models, LDA was coupled with variable selection algorithms. Two distinct approaches were tested to find the most parsimonious and mechanistically meaningful subset of variables: the SPA, a deterministic method that minimizes collinearity, and the GA, a stochastic evolutionary approach. Both algorithms aim not just to classify the samples but to isolate the most discriminant wavelengths/wavenumbers, providing a chemically informed explanation for the differentiation between the genera. The SPA was tested with variable selection windows (1–10) and GA with three tested combinations (population/generation: 100/100; 200/200; 300/300), 5% mutation, and 60% crossover. In each GA run, a maximum of 10 variables were selected. The Kennard–Stone method was used to divide the samples; 22 samples were selected for the training set (10 *Lupinus* and 12 *Mimosa*) and 11 samples for the test set (5 *Lupinus* and 6 *Mimosa*). The performance metrics were calculated using the training and test sets with the results for the training set being presented using cross-validation. To evaluate the LDA models, the following figures of merit were calculated: sensitivity (SEN) (eq 1), specificity (SPE) (eq 2), and accuracy (ACC) (eq 3), where TP means true positive, TN true negative, FP false positive, and FN false negative.^38^

1 Sensitivity(SEN)(%)=(TP/(TP+FN))×100

2 Specificity(SPE)(%)=(TN/(TN+FP))×100

3 Accuracy(ACC)(%)=((TP+TN)/(TP+FN+TN+FP))×100

where TP is the number of true positives (class A samples correctly classified as class A), TN is the number of true negatives (class B samples correctly classified as class B), FP is the number of false positives (class B samples incorrectly classified as class A), and FN is the number of false negatives (class A samples incorrectly classified as class B).^38^

## 3 Results and Discussion

The genera *Lupinus* and *Mimosa*, despite belonging to the same family (Fabaceae), are taxonomically and evolutionarily distant. *Lupinus* belongs to the subfamily Papilionoideae, the tribe Genisteae. *Mimosa* currently belongs to the subfamily Caesalpinioideae, tribe Mimoseae, *Mimosa* clade.^39^ Given their placement in different subfamilies and their distinct morphologies (both vegetative and reproductive), differences in the presence and structure of their secondary metabolites are expected. Previous studies on *M. tenuiflora*,^40^ *Mimosa artemisiana* Heringer and Paula,^41^ *Lupinus albus* L. and *Lupinus angustifolius* L.,^42^ and *L. lanatus* ^13^ have reported a diversity of secondary metabolites, including flavonoids (flavones, isoflavones, flavonols, flavanones, and isoflavanones) and alkaloids (indolic and quinolizidine), among others.

Our findings in this study corroborate reports in the literature, supporting the differentiation of the two genera based on their chemical composition.

### 3.1 Multivariate Analysis

The UV–vis and FTIR spectral profiles of the average values for the samples of the two genera studied (*Lupinus* and *Mimosa*) after pretreatments are shown in Figure 1. By visually examining the spectra obtained from this exploratory analysis, we already observe similarities and differences between the profiles of the different samples analyzed. During this initial exploratory and visual inspection of the spectral data (Figure 1) and subsequent PCA scores, no samples were identified as anomalous outliers. Consequently, no samples were excluded from the data set prior to model training. All samples were retained to accurately capture and reflect the natural biological and intraspecific variability inherent to the *Lupinus* and *Mimosa* species evaluated, thereby ensuring the development of robust and realistic predictive models.

**Figure 1:**
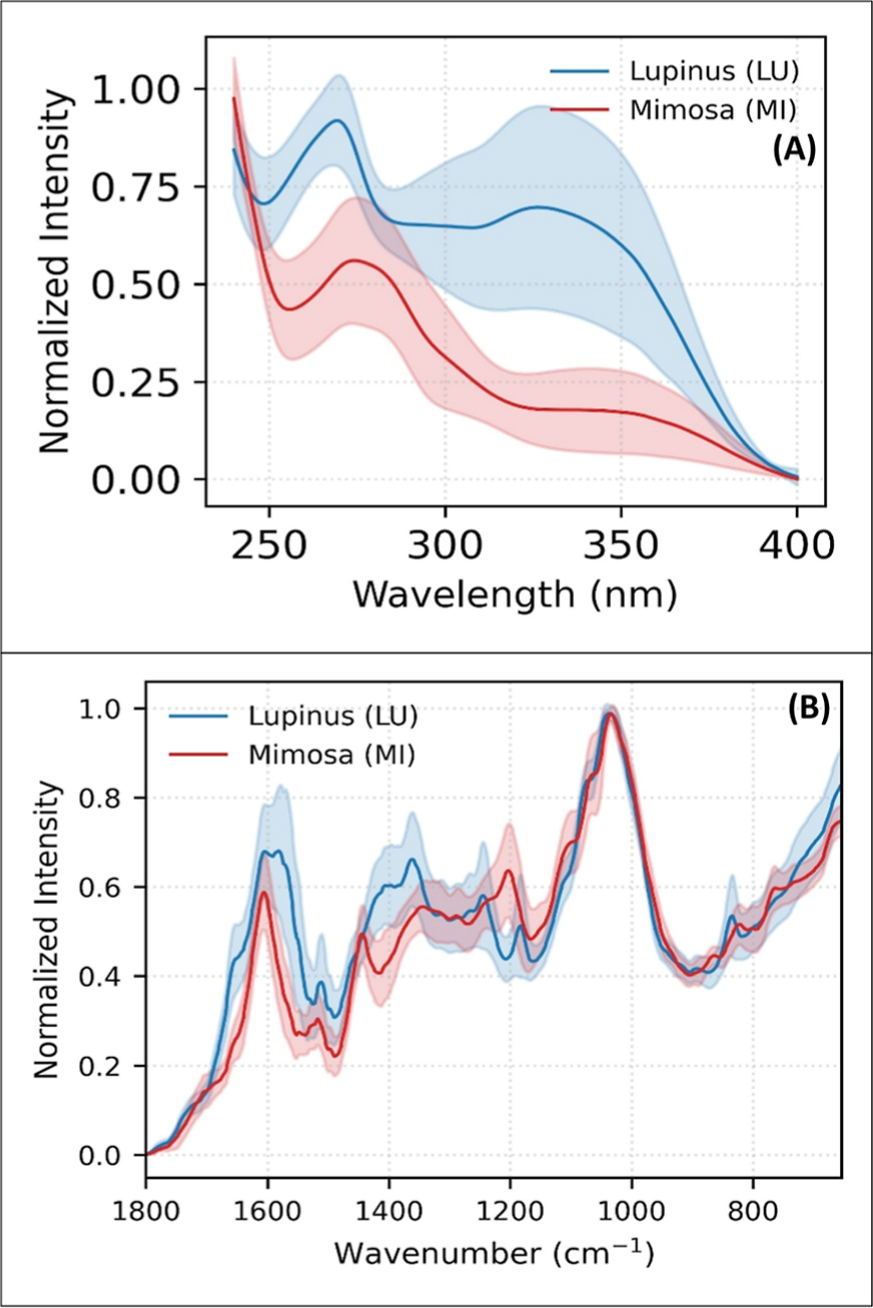
Average profiles obtained in the analyses by (A) UV–vis (240–400 nm) and (B) FTIR (1600–600 cm^–1^). Results obtained by the average of the analysis of all samples of each studied genera: LU: *Lupinus* and MI: *Mimosa*. Shaded areas represent the standard deviation (*n* = 100 for UV; *n* = 66 for FTIR).

#### 3.1.1 Unsupervised Methods

The HCA and PCA results allowed for the identification of two distinct groups. The dendrograms (Figure 2), scores and loadings of the principal components (Figures 3 and 4), support this observation.

**Figure 2:**
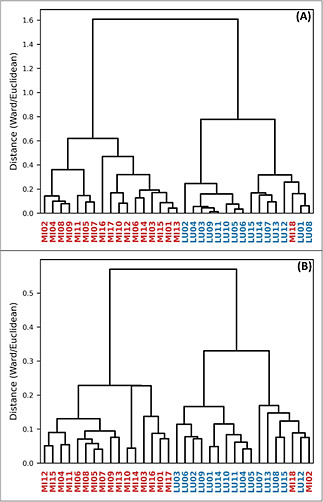
HCA-dendrogram (A): UV–vis and (B): FTIR. Results expressed by the average of the analysis of each sample, from the total set of the two genera: LU—*Lupinus* and MI—*Mimosa*.

**Figure 3:**
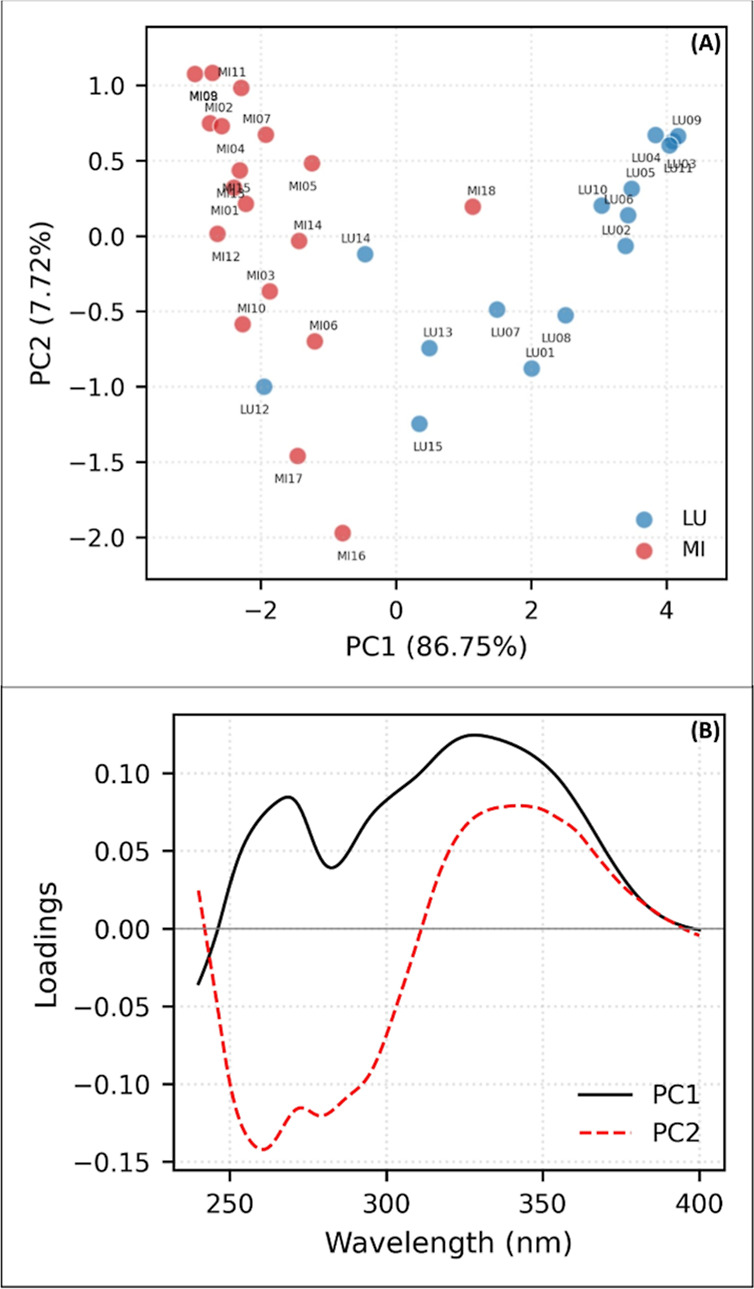
PCA-UV–vis (240–400 nm). (A) Scores and (B) loadings. Results expressed by the average of the analysis of each sample, from the total set of the two genera: LU—*Lupinus* and MI—*Mimosa*.

**Figure 4:**
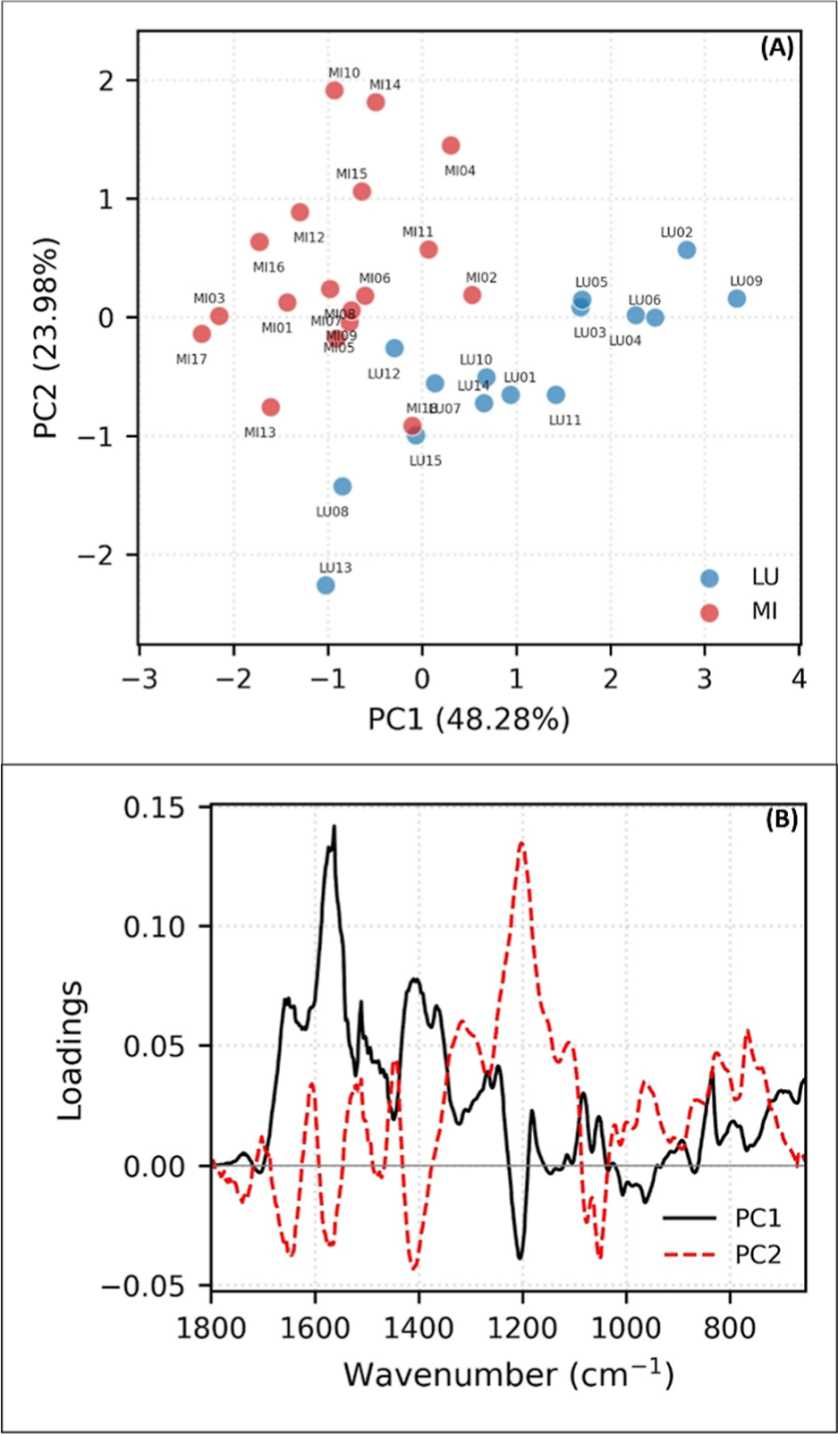
PCA-FTIR (1800–600 cm^–1^). (A) Scores and (B) loadings. Results expressed by the average of the analysis of each sample, from the total set of the two genera: LU—*Lupinus* and MI—*Mimosa*.

The principal components, PC1 and PC2, in both methods were able to explain and enable the visualization of strong patterns and relationships within the samples of the two studied genera. In the UV–vis analyses (Figure 3), PC1 was able to explain 86.75% of the variability, while PC2 explained 7.72%. For FTIR (Figure 4), these results were 48.28% (PC1) and 23.98% (PC2). By analyzing the PCA loading graphs, it is possible to observe the contribution of each original variable (wavelengths for UV–vis and wavenumbers for FTIR) to the PCs. There is a clear separation between the “LU” (blue) and “MI” (red) groups along PC1, suggesting that the original variables that contribute most to PC1 are those that best discriminate the two groups.

As shown in Figure 3, in PC1, the *Lupinus* samples are characterized by a predominance of positive signals, with more intense signals in the approximate regions between 259 and 269 and 340–350 nm. In contrast, the *Mimosa* samples show a predominance of negative signals, with more intense signals in the approximate region of 240 nm. In PC2, both genera exhibit both positive and negative signals.

Substitutions in the basic structure of flavonoids, such as hydroxylations or glycosylations, alter the wavelengths of maximum absorption in the UV–vis. Studies demonstrate that the number and position of hydroxyl groups in rings A and B modulate the absorption bands, ranging from 300 to 385 nm (Band II, Ring A) and 240–285 nm (Band I, Ring B).^43,44^

Similarly, Figure 4 shows that in PC1, *Lupinus* samples exhibit positive signals (with more intense signals in the approximate regions of 1580–1560 cm^–1^), and *Mimosa* samples exhibit negative signals (with more intense signals in the approximate regions of 1215–1200 cm^–1^). In PC2, positive signals are more related to *Mimosa* (with more intense signals around 1200 cm^–1^), while negative signals are more related to *Lupinus* (with more intense signals in the approximate regions of 1650, 1570, and 1410 cm^–1^).

FTIR spectroscopy reveals diagnostic wavenumber bands for identifying functional groups present in the structure of flavonoids. The regions between 1800–1540 cm^–1^, 1600–1300 cm^–1^, and 909–650 cm^–1^ correspond, respectively, to carbonyl groups (C=O), aromatic rings, and aliphatic structures. The region of 1300–909 cm^–1^, although complex and characteristic of each molecule, is mainly used for spectral comparison and identity confirmation.^45^

Because they have different chemical structures in their composition but belong to the same class of secondary metabolites (e.g., flavonoids and alkaloids), unsupervised exploratory analysis revealed a clear tendency for the two studied genera to separate into distinct groupings. This analysis also allowed for the visualization of wavelength and wavenumber regions found in the reported structures of both genera. Therefore, the natural separation observed in the PCA score plots is fundamentally driven by these specific spectral features: the variance in the UV–vis absorption bands (associated with distinct flavonoid substitutions) and the FTIR vibrational modes (especially the characteristic C=O stretching of quinolizidine alkaloids in *Lupinus* and the C–O vibrations of specific flavonoids in *Mimosa*). This demonstrates that the discrimination is not merely a mathematical artifact but a direct reflection of their underlying chemical differences.

Based on the visualization and understanding of the data structure obtained through exploratory analysis (HCA and PCA), we performed a LDA to test whether these groups are significantly distinct.

#### 3.1.2 Supervised Methods

##### 3.1.2.1 Linear Discriminant Analysis (LDA): Successive Progression Algorithm (SPA) and Genetic Algorithm (GA)

Analysis of the averaged UV–vis (Figure 1A) and FTIR (Figure 1B) spectra reveals similarities between the genera *Lupinus* and *Mimosa*, which aligns with the literature describing flavonoids and alkaloids as the main metabolites present in both.^14,46^ Despite the similarities, subtle differences in absorption patterns, such as peak shifts and variations in band intensity, indicate variations in the chemical composition between the genera.

Analyses with variable selection of spectral data (SPA-LDA and GA-LDA) allowed the identification of distinct groups corresponding to genera *Lupinus* and *Mimosa*, demonstrating excellent performance in sample classification.

The results of the figures of merit, calculated from eqs 1–3, to test the LDA models were all satisfactory, with values between 95 and 100% for all figures (sensitivity, specificity, and accuracy).

The best results obtained by SPA-LDA and GA-LDA for the UV–vis data are presented in Figure 5. The best result for GA-LDA was obtained with the following combination: population: 100; generation: 100; mutation 5%; crossover 60%. Figure 5A,B shows the variables selected for each algorithm tested, with one variable in SPA and four variables in GA, respectively. The Fisher weight plots (Figure 5C,D) demonstrate the contribution of the selected variables to each genus studied (256 nm in SPA; 257 and 356 nm in GA, related to *Lupinus*, and 284 and 261 nm in GA, related to *Mimosa*). The discriminant function graphs (Figure 5E,F) demonstrate that the samples were categorized with an excellent correct classification rate, with 100% in the training (circles) and test (stars) sets for the two algorithms tested.

**Figure 5:**
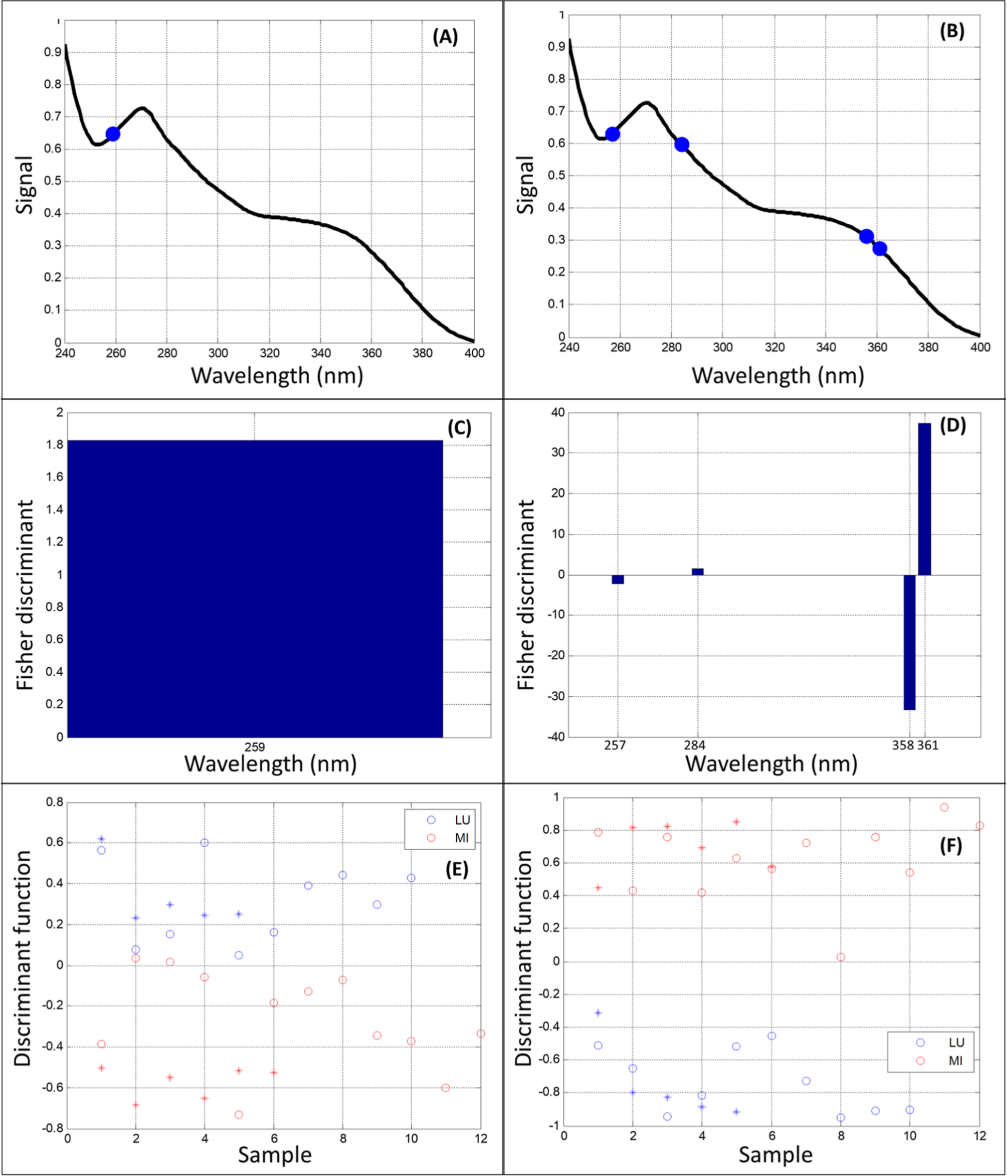
UV–vis results: SPA-LDA (A,C,E); GA-LDA (B,D,F). (A,B) Variable selection in the two tested algorithms; (C,D) Fisher weights with wavelengths selected in the two tested algorithms; and (E,F) training sets (circles) and test sets (stars) with the separation of the two analyzed classes, in the two tested algorithms. LU: *Lupinus* and MI: *Mimosa*.

Figure 6 shows the best results obtained by SPA-LDA and GA-LDA for the FTIR data. The best result for GA-LDA was obtained with the combination: population: 100; generation: 100; mutation 5%; and crossover 60%. Figure 6A,B shows the variables selected for each algorithm tested with six variables in SPA (Figure 6A) and nine variables in GA (Figure 6B). The samples were also categorized with an excellent correct classification rate, with 100% in the training (circles) and test (stars) sets for both algorithms tested (Figure 6E,F). Finally, the contribution of the selected variables to each genus studied can be observed in Figure 6C,D (1658, 1595, and 665 cm^–1^ in SPA; 1476, 1105, 828, 791, and 660 cm^–1^ in GA, related to *Lupinus*, and 1561, 1185, and 1103 cm^–1^ in SPA; and 1535, 1107, 781, and 675 cm^–1^ in GA, related to *Mimosa*).

**Figure 6:**
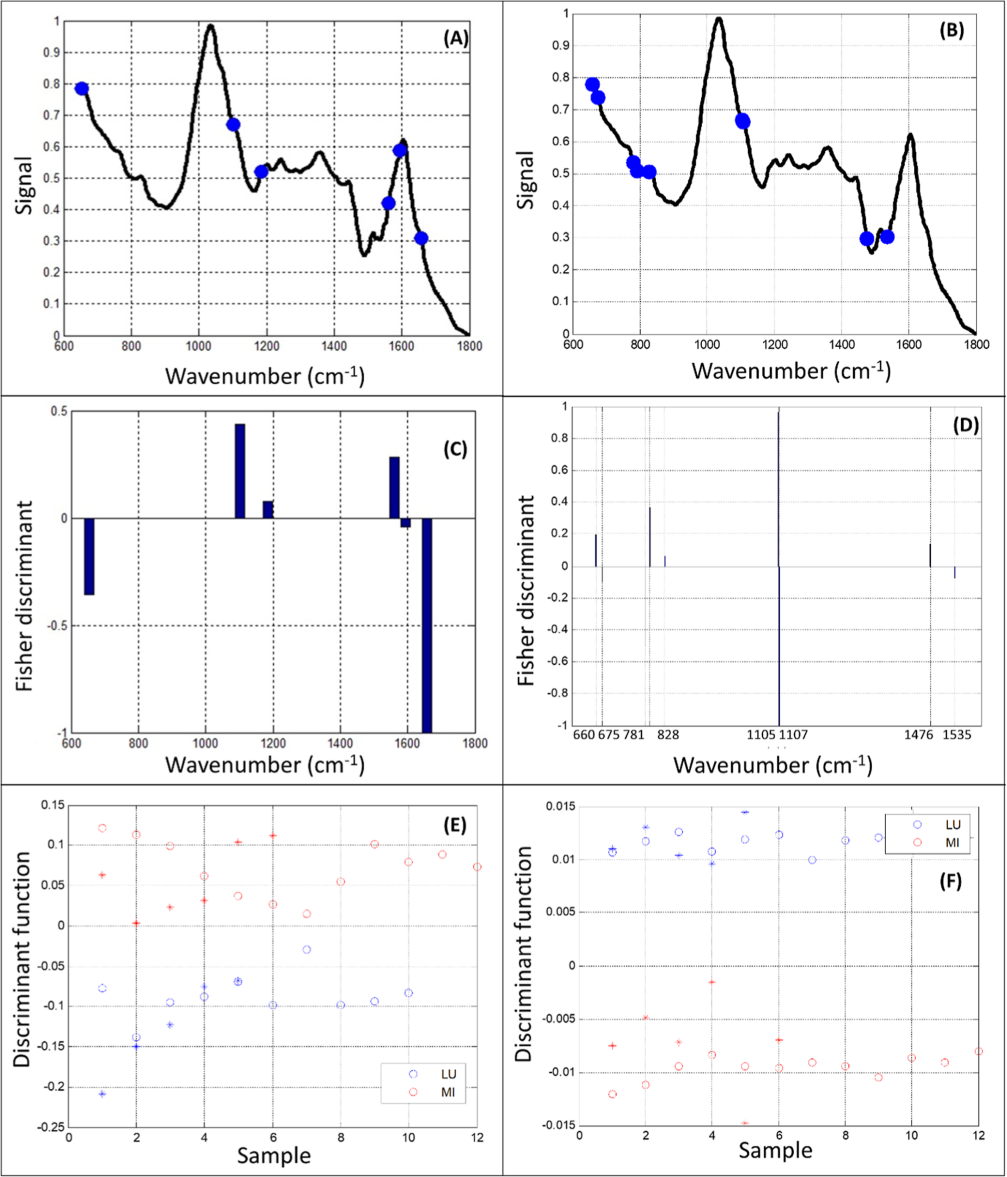
FTIR results: SPA-LDA (A,C,E); GA-LDA (B,D,F). (A,B) Variable selection in the two tested algorithms; (C,D) Fisher weights with wavenumber selected in the two tested algorithms; and (E,F) training sets (circles) and test sets (stars) with the separation of the two analyzed classes, in the two tested algorithms. LU: *Lupinus* and MI: *Mimosa*.

Despite the excellent classification metrics achieved, it is important to briefly outline the applicability domain of the proposed models. The current predictive models were developed and validated using species collected specifically in the Rio Grande do Sul region (Table 1). Since plant secondary metabolism can be influenced by geographic, climatic, and seasonal variations, the direct application of these specific models to *Lupinus* and *Mimosa* species from entirely different biomes may require prior model updating or recalibration. Furthermore, although the models successfully discriminate between these two genera, their applicability does not currently extend to identifying individual species within the genera, which remains a limitation to be addressed in future studies with larger sample sizes.

The similarity in wavelengths (Figures 5A and 6A) and wavenumbers (Figures 5B and 6B) selected by the algorithms to classify/separate the genera *Lupinus* and *Mimosa* reflects the presence of secondary metabolites from common classes but with structural differences.

To provide a chemical rationale for the discrimination achieved by the LDA models, the most significant variables selected by SPA and GA were tentatively assigned to specific functional groups and secondary metabolites known to occur in these genera. This correlation confirms that the mathematical models are grounded in the distinct biosynthetic pathways of each genus. Table 2 summarizes these spectral assignments, linking the mathematical markers to the underlying chemical profiles of *Lupinus* (notably quinolizidine alkaloids) and *Mimosa* (rich in diverse flavonoids).

**Table 2:** Tentative Assignment of Key Spectral Variables Selected by SPA-LDA and GA-LDA for the Differentiation of *Lupinus* and *Mimosa* Genera

technique	selected variable	tentative assignment	chemical relevance	refs
UV–vis	∼259 nm	Band II (Ring A) absorption of isoflavones	presence of genistein and derivatives in *Lupinus*	^42,43^
UV–vis	284 nm	Band II absorption/phenolic B-ring	variation in hydroxyl/methoxyl substitutions	^43,44^
UV–vis	356–361 nm	Band I (Ring B) absorption of flavonols	flavones and flavonols (e.g., quercetin) in *Mimosa*	^41,44,47^
FTIR	1658 cm–^1^	C=O stretching (Amide I)	carbonyl groups in quinolizidine alkaloids	^13,45^
FTIR	1103–1107 cm^–1^	C–Ostretching vibrations	flavonoid glycosides and phenolic backbones	^45^

Previous studies have identified acylated and glycosylated derivatives of myricetin and quercetin in *M. pigra*,^47^ while *M. artemisiana* revealed the presence of these flavones, in addition to hydroxylated and dimethoxylated derivatives of kaempferol.^41^ The aglycones of these flavonols exhibit maximum absorption bands in the 360 nm region, attributed to ring A, with variations resulting from substitutions in this ring. This signal, therefore, suggests that it is a significant marker for the *Mimosa* genus. Additionally, the detection of gallic acid and catechin in *Mimosa pudica* and *M. tenuiflora*,^40,48^ compounds with maximum absorption near 280 nm (Ring B), reinforces the importance of the 284 nm variable (lowest intensity) in the discrimination between the genera. The variations observed around 280 nm are associated with substitutions by hydroxyl, methoxyl, or glycoside groups.^43,44^

Findings from other studies reported the presence of the isoflavone genistein in *L. albus* and *L. angustifolius*, with a maximum absorption wavelength at 259 nm.^42^ Additionally, quinolizidine alkaloids, identified in *L. lanatus*, also showed absorption in this spectral region.^13^ These findings corroborate the results of the present study and emphasize the relevance of the selected variable for the classification of genera *Lupinus* in relation to genus *Mimosa*.

Fisher weight plot analysis of FTIR spectra (Figure 6C,D) revealed signals suggestive of clusters relevant to the discrimination between genera *Lupinus* and *Mimosa*. For *Lupinus*, the wavenumber 1658 cm^–1^ (C=O) was selected as significant, which is strongly suggestive of the presence of carbonyls typically found in quinolizidine alkaloids, which is characteristic of the genus. In *Mimosa*, the wavenumbers 1103 and 1107 cm^–1^ (C–O), observed in SPA and GA, respectively, are tentatively assigned to stretching vibrations of C–O bonds, which is consistent with the abundance of flavonoids reported for this genus.^45^

Despite the presence of similar classes of secondary metabolites, which explains the proximity of UV–vis absorption wavelengths and FTIR chemical shifts for both genera included in this study, the combination of simple analytical techniques and chemometrics allowed the correct classification of all species. The high accuracy and sensitivity of the applied chemometric methods (LDA with successive progressions and GAs) for both analytical methods (UV–vis and FTIR) demonstrate the effectiveness of the approach. Cross-validation and performance metrics confirm the robustness of the models, validating LDA as an effective tool for complex data analysis without the need for advanced techniques such as NMR or chromatography.

## 4 Conclusion

Our study demonstrated the effectiveness of UV–vis and FTIR spectroscopy, combined with variable selection algorithms, in distinguishing species from the genera *Lupinus* and *Mimosa* collected in Rio Grande do Sul, Brazil. Subtle spectral variations, which are tentatively assigned to differences in specific secondary metabolites (such as quinolizidine alkaloids and flavonoids), allowed accurate discrimination. The coupling of LDA with the SPA and GA proved to be essential to overcome data collinearity and identify mechanistically meaningful spectral markers, yielding highly accurate predictive models. While future studies encompassing broader geographic regions and larger sample sizes are necessary to expand the applicability domain, this research highlights the potential of machine learning-assisted spectroscopy as a robust auxiliary tool in plant taxonomy, complementing traditional morphological analyses.

## Notes

During the preparation of this work, the author used the AI tool Gemini 2.5 Flash to improve the writing and language of the manuscript. After using this tool, the author reviewed and edited the content as necessary and assumes full responsibility for the content of the published article.
